# Virtual Reality–Based Program for Pediatric Patients With Amblyopia: Protocol for a Multicenter, Randomized, Open-Label, Two-Arm Study

**DOI:** 10.2196/85194

**Published:** 2026-01-21

**Authors:** Ken Nagino, Yuichi Okumura, Masakazu Hirota, Saiko Matsumura, Tadashi Matsumoto, Takashi Negishi, Akie Midorikawa-Inomata, Makiko Ui, Takao Hayashi, Yuichi Hori, Shintaro Nakao, Takenori Inomata

**Affiliations:** 1 Department of Hospital Administration, Juntendo University Graduate School of Medicine, Tokyo, Japan; 2 Department of Telemedicine and Mobile Health, Juntendo University Graduate School of Medicine, Tokyo, Japan; 3 InnoJin, Inc, Tokyo, Japan; 4 Department of Ophthalmology, Juntendo University Graduate School of Medicine, 2-2-1 Hongo, Bunkyo-ku, 113-0033, Japan, +81-3-3813-3111; 5 Department of Orthoptics, Faculty of Medical Technology, Teikyo University, Tokyo, Japan; 6 Department of Ophthalmology, School of Medicine, Teikyo University, Tokyo, Japan; 7 Graduate Degree Program of Health Data Science, Teikyo University, Tokyo, Japan; 8 Department of Ophthalmology, Toho University Faculty of Medicine, Tokyo, Japan; 9 CS Eye Clinic, Tokyo, Japan

**Keywords:** amblyopia, dichoptic training, pediatric, randomized controlled trial, treatment adherence, virtual reality, digital therapeutics, software as a medical device, telemedicine, gamification, hand-eye coordination

## Abstract

**Background:**

Conventional amblyopia treatment involves the occlusion of the fellow eye using an eye patch. However, this approach imposes a substantial psychological and physical burden on pediatric patients with amblyopia, resulting in low adherence and suboptimal visual outcomes. As responsiveness to therapy declines beyond early childhood, treatments that can enhance adherence and improve efficacy are needed. We developed a virtual reality dichoptic training app (VR app) that integrates gamification and hand-eye coordination training in pediatric patients with amblyopia.

**Objective:**

This study investigated the effect of a VR app on visual acuity improvement in pediatric patients with amblyopia compared with conventional occlusion therapy using an eye patch.

**Methods:**

This is a multicenter, open-label, prospective, randomized controlled trial that will be conducted at 3 centers in Tokyo, Japan. Pediatric patients aged 3-10 years with anisometropic, strabismic, or refractive amblyopia will be enrolled. Participants will be assigned 1:1 to a VR app group or a control group. The VR app group will use the VR app at home for 1 hour per day for 6 months. The control group will undergo conventional occlusion therapy with an eye patch for 6 months. Each group will comprise 15 participants (30 participants in total). Ophthalmologic examinations will be performed at baseline and at weeks 4, 8, 12, 16, 20, and 24. The primary endpoint will be the change in the best-corrected visual acuity in the amblyopic eye from baseline to week 12. Secondary endpoints will include changes in best-corrected visual acuity, stereopsis, and ocular deviation through week 24. We will also assess treatment adherence, defined as the ratio of the cumulative actual treatment time to the cumulative prescribed time; record adverse events, and evaluate usability in the VR app group. Longitudinal changes in outcome measures will be analyzed using a mixed-effects model.

**Results:**

Participant enrollment will start from January 1, 2026, to September 30, 2026. The data analysis will begin on October 1, 2026, and the results will be reported by March 31, 2027.

**Conclusions:**

This study will clarify the effectiveness of the newly developed VR app in improving treatment outcomes and adherence among pediatric patients with amblyopia. By addressing the limitations of conventional occlusion therapy and providing a potentially more efficacious and acceptable treatment option, the VR app may enhance clinical outcomes and represent a paradigm shift in the treatment of pediatric amblyopia.

## Introduction

Amblyopia is primarily characterized by reduced visual acuity in one or both eyes, with a prevalence of approximately 1% to 5% among children worldwide and an estimated prevalence of 0.1% to 0.2% in Japan [1-5]. Pediatric amblyopia is most commonly attributable to refractive errors, anisometropia, strabismus, and visual deprivation [6]. The treatment of amblyopia involves the use of fully corrective glasses in combination with occlusion therapy using an eye patch to train the amblyopic eye. However, the sensitive period of visual development extends only until approximately the age of 7 years [7], after which the effectiveness of treatment becomes significantly limited. Accordingly, amblyopia necessitates public health strategies aimed at early detection, the establishment of effective therapeutic approaches, and efficient management [8].

In Japan, under the Maternal and Child Health Law, all 3-year-old children undergo ophthalmologic screening, including testing for amblyopia, as part of their routine health examination [9]. The screening process is implemented in successive stages. Initially, parents conduct vision testing at home, and the results are submitted to the health examination site. At the venue, pediatricians, in collaboration with public health nurses and other nonophthalmic professionals, review the submitted data and, when indicated, perform supplementary preliminary assessments. If abnormalities are suspected, the child is subsequently referred to an ophthalmology clinic, where ophthalmologists conduct comprehensive examinations [9]. Nevertheless, reports indicate that home-based vision screening overlooks approximately 24.2% of pediatric patients with amblyopia [9]. According to a report assessing vision screening at 30-35 months of age in the United Kingdom, orthoptist-administered screening achieved sensitivities and specificities approaching 100%. Contrastingly, screening performed by healthy visitors yields a sensitivity of only approximately 50%, with approximately half of the affected cases remaining undetected [10]. Accordingly, evidence suggests that certain cases of amblyopia go unrecognized when comprehensive examinations at medical institutions are not undertaken, thereby precluding the timely initiation of appropriate therapy [10].

In occlusion therapy utilizing an eye patch, whereby the healthy eye is covered to promote the use of the amblyopic eye, the duration of daily occlusion must be extended with advancing patient age, thereby imposing an increasing psychological and physical burden on the child [11,12]. Excessive burden associated with amblyopia treatment may result in reduced adherence, manifested by noncompliance with the occlusion of the healthy eye by the child or premature discontinuation of therapy by parents of their own judgment, thereby attenuating the overall effectiveness of amblyopia management [13-15]. A study assessing adherence to occlusion therapy using an eye patch reported that patients complied with the patch wear for approximately half of the prescribed occlusion time [16]. Adherence to amblyopia therapy has been demonstrated to exhibit a dose-response relationship with therapeutic efficacy [17]. Accordingly, the development of alternative modalities to occlusion therapy that lessen the psychological and physical burden, thereby mitigating the decline in adherence, is warranted.

**Figure 1.**
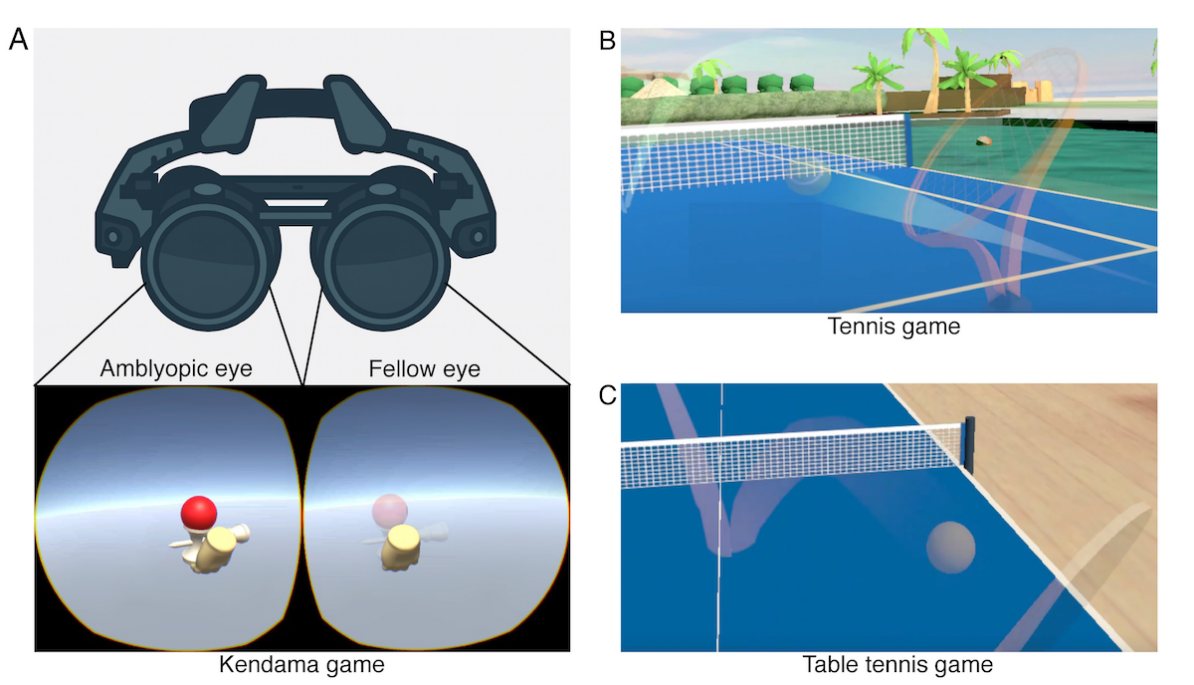
The virtual reality (VR) app for pediatric patients with amblyopia. (A) VR head–mounted display and a screenshot of the Kendama game projected to each eye. In the fellow eye, the ball is displayed as semitransparent. (B) Screenshot of the tennis game projected to the fellow eye. (C) Screenshot of the table tennis game projected to the fellow eye.

To overcome the challenges associated with pediatric amblyopia therapy, we developed a clinical investigational device, the virtual reality (VR) app, which utilizes VR as a programmatic medical device for amblyopia training in children (Figure 1A) [18]. The VR-based Software as Medical Device (SaMD) for pediatric amblyopia incorporates functionality to partially render the visual input to the healthy eye transparent and includes a gaming module operated through hand-tracking controllers [18]. The VR app enables amblyopia training through interactive gameplay, wherein part of the image projected onto a healthy eye is rendered transparent to facilitate the engagement of the amblyopic eye during the task [18]. An illustrative example of a VR-based SaMD for pediatric amblyopia training is the Luminopia One (Luminopia, Inc.), which was granted authorization by the U.S. Food and Drug Administration [19]. The therapeutic effect of Luminopia One is achieved by attenuating the contrast of visual stimuli presented to the healthy eye, which in turn promotes the use of the amblyopic eye and enhances the efficacy of amblyopia training [19]. The principal difference between Luminopia One and the VR app is that Luminopia One delivers only contrast-modified visual content, whereas the VR app integrates interactive games requiring active physical engagement via hand-tracking controllers, such as kendama (Figure 1A), tennis (Figure 1B), and table tennis (Figure 1C) [18,19]. In contrast to the amblyopia training implemented in Luminopia One, which is limited to passive viewing of visual content with the amblyopic eye, the VR app incorporates VR-based games that require active hand-eye coordination, potentially conferring greater therapeutic efficacy in amblyopia treatment [20]. Thus, the VR app–based amblyopia training may confer greater therapeutic efficacy than conventional approaches, owing to its distinctive advantages: alleviation of psychological and physical burden by obviating the use of an eye patch, improvement of adherence through gamification, and provision of effective training that incorporates hand-eye coordination [17,18,20,21].

This study aims to conduct a multicenter clinical trial to assess the therapeutic effectiveness of a VR app in pediatric amblyopia. The use of the VR app is expected to enable amblyopia training with higher adherence and enhanced efficacy compared to conventional occlusion therapy, with the potential to mitigate the societal burden attributable to pediatric amblyopia.

## Methods

### The VR Dichoptic Training App for Pediatric Patients With Amblyopia

A VR app for pediatric patients with amblyopia was developed by InnoJin Inc., ImaCreate Co. Ltd., and Sumitomo Corporation. The VR app has not yet been approved as an SaMD in Japan as of August 31, 2025. After being approved for SaMD, pediatric patients with amblyopia will be able to download and use the VR app for pediatric amblyopia on a VR device after receiving a prescription from their physicians.

The VR app for pediatric patients with amblyopia uses a gamification approach to train the amblyopic eye in pediatric patients. Currently, the VR app for pediatric patients with amblyopia provides kendama, tennis, and table tennis games (Figure 1A-C, respectively). Kendama is a children’s activity that uses the traditional Japanese skill toy called “kendama” [22], which consists of a handle (called “ken,” meaning “sword”) and a ball (called “tama” or “dama”) connected by a string. The basic way to play is to catch the thrown ball with 1 of the 3 cups on the handle, or to “spike” the ball by inserting the tip of the handle into a hole on the ball. Countless combinations and styles exist to catch the ball, and players often combine techniques in unique ways to perform tricks. In the VR app, players will wear the VR head–mounted display and swing the controllers, allowing them to move the kendama handle and catch the ball in a virtual world. The “tennis” and “table tennis” games will both be sports in which players hit a ball back and forth with a racket by swinging the controllers to move a racket and hit the ball in the virtual world. This study will use the MeganeX Superlight 8K (Shiftall Inc.) as the head-mounted display.

The VR app will provide amblyopia training within the games. By ensuring that the ball image projected to the healthy eye is semitransparent or transparent, the program replicates the occlusion of the healthy eye and encourages the use of the amblyopic eye while playing the game. Additionally, the VR app for pediatric patients with amblyopia will incorporate hand-eye coordination training using hand-tracking controllers, which may promote the greater development of visual pathways compared with conventional approaches that require only image viewing without physical movement [20].

### Study Setting

This multicenter, open-label, randomized controlled trial is scheduled to be conducted from January 1, 2026, to March 31, 2027. Patients will be enrolled at 3 medical institutions: Toho University Omori Medical Center (Tokyo, Japan), Teikyo University Hospital (Tokyo, Japan), and the CS Eye Clinic (Tokyo, Japan).

### Ethical Considerations

This study has been approved by the Teikyo University Certified Review Board, Tokyo, Japan (protocol version 1.0; August 14, 2025; approval number: C0130-6). The parents of the participating children, acting as their representatives, will provide written informed consent. Informed assent will also be obtained whenever possible from children judged to have at least an elementary school level of understanding. All individuals involved in this study will make every effort to protect the personal information and privacy of the participating patients, and patient data will be anonymized to ensure that no individual can be identified. Participants who complete all study procedures through the final visit at week 24 will be provided with a voucher valued at 20,000 yen (approximately US $128, as of January 5, 2026). Participants who discontinue participation prior to the final visit, after completing at least 12 weeks but fewer than 24 weeks of study participation, will be provided with a voucher valued at 10,000 yen (approximately US $64, as of January 5, 2026).

### Inclusion and Exclusion Criteria

Outpatients who visit the collaborating study institutions will be recruited and must meet the following inclusion criteria: between 3 and 10 years old at time of enrollment; diagnosis of anisometropic amblyopia, strabismic amblyopia, or refractive amblyopia; best-corrected visual acuity (BCVA) of 0.3 logMAR or worse in the amblyopic eye at screening; and have a BCVA in the fellow eye of 0.2 logMAR or better at screening [19]. The inclusion age range was chosen because amblyopia treatment responsiveness declines with age, and improvement is less likely in older children. In this study, limiting enrollment to younger children was considered ethical because the VR-based intervention’s effectiveness remains uncertain.

In this study, amblyopia is confirmed before enrollment, and since anisometropic and refractive amblyopia may occur even with small interocular acuity differences, we did not require a 2-line difference as eligibility criterion, though commonly referenced as a diagnostic indicator of amblyopia [23]. The exclusion criteria will be as follows: inability to obtain consent from the legal guardian; where applicable, inability to obtain assent from the patient; history of intraocular surgery or refractive surgery; history of photosensitive epilepsy; inability to comply with the study protocol due to psychological, familial, or geographic reasons; or inability to perform hand-eye coordination tasks, as determined by physical or mental conditions or by observation during a trial use of the study device, indicating a clear inability to establish hand-eye coordination.

### Patient Withdrawal

Patients will be withdrawn from the study if, due to the occurrence of adverse events, it becomes difficult to continue the research; if patients or their parents or guardians request to end their participation; if the research study is discontinued; or if the principal investigator and subinvestigators judge it appropriate to discontinue the research.

### Study Procedures

**Figure 2.**
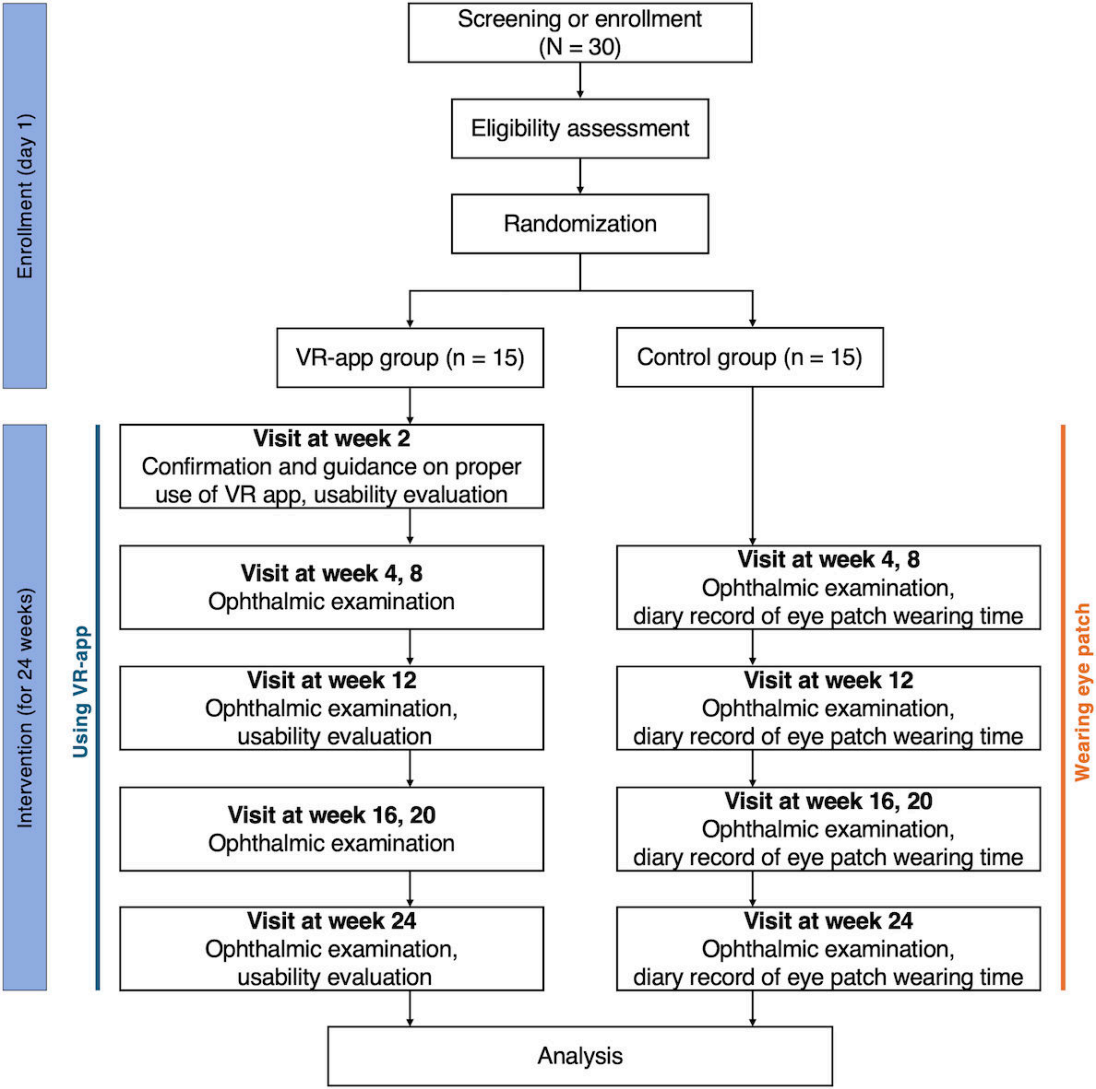
Study flowchart. VR: virtual reality.

Figure 2 illustrates the study enrollment procedure. After obtaining informed consent, pediatric patients will be randomly assigned to either a VR app group, which will undergo amblyopia treatment using the VR app for pediatric patients with amblyopia combined with full refractive correction glasses, or a control group, which will undergo amblyopia treatment based on occlusion treatment with an eye patch combined with full refractive correction glasses [24]. Patients assigned to the VR app group will use the VR app for amblyopia at home for 1 hour per day for 6 months, in combination with full refractive correction glasses. Patients assigned to the control group will follow occlusion therapy with an eye patch for 6 months, also wearing full refractive correction glasses, and the duration of eye patch use will be determined by the prescribing physician. Both groups underwent ophthalmological examinations on the first day of the trial and at weeks 4, 8, 12, 16, 20, and 24. Ophthalmological examinations will include measurements of visual acuity, intraocular pressure, strabismus angle, stereopsis, refractive error, and corneal curvature. Only the VR app group will perform a digital stereopsis test using the VR app once per week and complete a usability questionnaire on the VR app at weeks 2, 12, and 24. The week 2 visit conducted only for the VR app group is intended to confirm that the study participants, particularly those unfamiliar with VR devices, are using the VR app correctly and facilitate their continued appropriate use. Only the control group will record their daily duration of eye patch use in a diary. Study participation will end for both groups after the 24-week visit.

### Data Collection and Outcome Assessments

The data collection items are listed in Textbox 1. Data on patient characteristics, ophthalmological examination results, VR app malfunctions, and adverse events will be collected from both the VR app and control groups. The daily usage time of the VR app, stereopsis test results obtained via the VR app, and usability evaluation results were collected only for the VR app group. Daily eye patch usage time will be collected only in the control group.

Visual acuity will be measured by BCVA using the Landolt-C chart at a distance [25]. Intraocular pressure will be measured using a noncontact tonometer [26]. The strabismus angle will be assessed using the alternate prism cover test [27]. Stereopsis will be evaluated using the Titmus stereo test [28]. The refractive error and corneal curvature radius will be measured using an autorefractor keratometer [29]. Daily VR app usage time will be calculated from the VR app usage logs, and daily eye patch wearing time will be reported by the patient’s parent in a diary.

Textbox 1. **Data collection.**

**Patient characteristics**

- Age, sex, medical history (including anisometropic amblyopia, strabismic amblyopia, and refractive amblyopia), and duration of treatment for amblyopia

**Ophthalmological examinations**

- Visual acuity, intraocular pressure, angle of strabismus, stereopsis test, refractive error, corneal curvature

**VR app group only**

- Daily virtual reality (VR) app usage time
- VR app–based stereopsis test
- Usability evaluation

**Control group only**

- Daily eye patch usage time

**Other outcomes**

- VR app failure
- Adverse events

### VR App–Based Stereopsis Test

**Figure 3.**
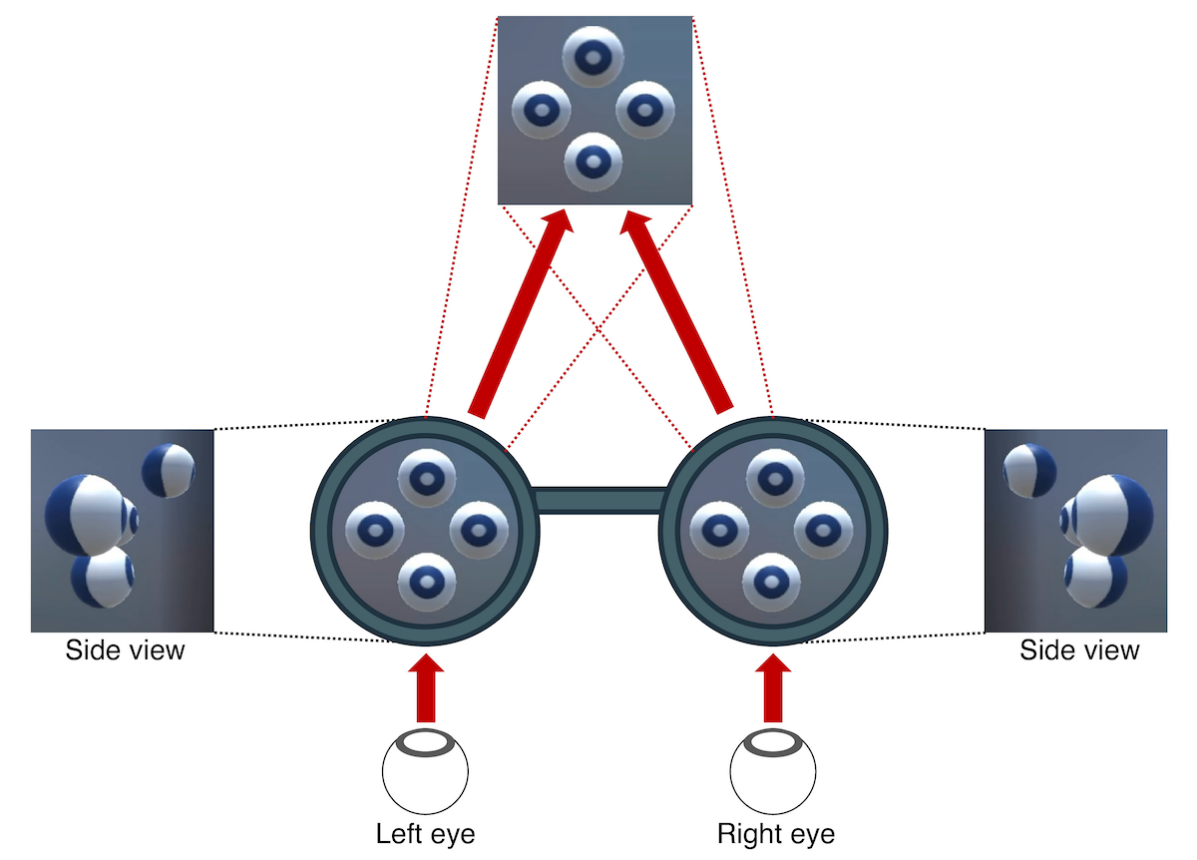
Virtual reality (VR) app–based stereopsis test. One of 4 spheres is moved forward along a path perpendicular to the viewing direction, and participants are asked to identify the moving sphere. To prevent detection of movement based solely on changes in size, the sphere is rendered such that its size decreases when approaching the viewer and increases when receding.

In the VR app group, a stereopsis test will be conducted using the VR app (Figure 3). In this test, 4 spheres are projected to both eyes, with 1 sphere placed directly in front of each eye. One of the 4 spheres will be moved forward along a path perpendicular to the viewing direction, and the participants will be asked to identify which sphere moved. When the sphere moves closer, its size decreases, whereas when it moves farther away, its size increases. This feature prevents the detection of sphere movement based solely on changes in size and ensures that the movement cannot be recognized by a single eye on the side of the moving sphere. This stereotest is a newly developed measure that has not yet undergone formal validation. The results of the VR app–based stereopsis test will be compared with those of the Titmus stereo test [28].

### Usability Evaluation

The parents and guardians of patients will respond to a usability questionnaire consisting of 15 fixed-choice statements regarding the VR app. This questionnaire was custom-designed for this study by selecting items from previously published questionnaires that were applicable to smartphone-based apps [30-32] and by adding original items relevant to the present VR app. Therefore, it has not been previously validated and was used for exploratory or descriptive purposes.

The questionnaire will cover 3 domains: game display (8 items), instruction manual (3 items), and overall impression of the parents (4 items), as shown in Textbox 2. In the game display domain, items will evaluate the clarity of background and text colors, the legibility of on-screen text size, the comprehensibility of on-screen text, the visibility of the size of displayed buttons, the comprehensibility of button labels or icons, the recognizability of button locations, the simplicity of operation, and the appropriateness of sound volume. In the instruction manual domain, items will examine the legibility of the manual text, the clarity of the manual content, and the clarity of guidance on operating the VR app. In the overall impression of the parents’ domain, items will assess children’s speed and ease of learning to operate the VR app, children’s ability to use it independently soon after instruction, children’s willingness to continue treatment using the VR app, and parents’ willingness for their child to continue the treatment using the VR app. Each question will be rated using a 5-point Likert scale (1, strongly disagree; 2, disagree; 3, neutral; 4, agree; and 5, strongly agree) [32,33].

Textbox 2. **Usability evaluation questionnaire.**

**Game display domain**

1. The colors of the background and text were easy to see.
2. The size of the displayed text was easy to read.
3. The meaning of the displayed text was easy to understand.
4. The size of the displayed buttons was easy to see.
5. The meaning of the displayed labels or icons of buttons was easy to understand.
6. The location of the displayed buttons was easy to recognize.
7. The operation was simple.
8. The sound volume was appropriate.

**Instruction manual**

1. The text size in the manual was easy to read.
2. The content of the manual was easy to understand.
3. The manual clearly explained how to operate the VR app.

**Overall impression of the parents**

1. The child was able to learn how to operate the VR app quickly.
2. The child was able to use the VR app independently soon after learning.
3. The child would like to continue treatment using the VR app.
4. The parent would like the child to continue treatment using the VR app.

### Adherence Evaluation

Treatment adherence will be evaluated for VR app usage and eye patch wearing, defined as the ratio of the actual treatment time to the target treatment time. In the VR app group, the target daily treatment time will be 1 hour of VR app usage per day, and the ratio will be calculated by dividing the actual VR app usage time by the total target treatment time (number of participation days in the study multiplied by 1 h). In the control group, the sum of the daily eye patch wearing times prescribed by the physician will constitute the target treatment time. This ratio will be calculated by dividing the actual eye patch wearing time by the total target treatment time throughout the study period.

### Primary Outcome

The primary outcome measure will be that the improvement in BCVA in the amblyopic eye from enrollment to 12 weeks in the VR app group is noninferior to the improvement in BCVA in the amblyopic eye from enrollment to 12 weeks in the control group. This outcome measure will be designed to confirm that the amblyopia treatment effect of the VR-based program is not inferior to that of conventional eye patch therapy. In addition, since previous studies investigating amblyopia treatment using a VR headset have shown a significant treatment effect following 12 weeks of use, comparing BCVA at 12 weeks will enable an efficacy assessment of the VR-based program for amblyopia under standard amblyopia treatment conditions, and thus, the 12-week time point will be adopted [19,34].

### Secondary Outcomes

Secondary outcomes are as follows:

1. Comparison of BCVA in the amblyopic eye at baseline and at weeks 4, 8, 12, 16, 20, and 24 between and within the control group (eye patch for the sound eye) and the VR app group
2. Comparison of stereopsis in the amblyopic eye at baseline and at weeks 4, 8, 12, 16, 20, and 24 between and within the control and VR app groups
3. In the VR app group, stereopsis in the amblyopic eye measured using the VR-based program for pediatric amblyopia will be compared with stereopsis measured using the Titmus stereo test at baseline and at weeks 4, 8, 12, 16, 20, and 24 during clinic visits
4. Comparison of the ratio of total wearing time to target wearing time (treatment adherence) at weeks 4, 8, 12, 16, 20, and 24 between the control and VR app groups
5. Comparison of the angle of strabismus in the amblyopic eye at baseline and at weeks 4, 8, 12, 16, 20, and 24 between the control and VR app groups
6. In the VR app group, the association between the VR gameplay status (including game type, play date/time, play duration, and game settings) and BCVA in the amblyopic eye, stereopsis, the ratio of total wearing time to the target wearing time, and ocular deviation will be evaluated
7. Malfunctions of the VR-based program for pediatric amblyopia
8. Adverse events
9. Usability evaluation (VR app group only)

To evaluate the effects of amblyopia treatment in the control and VR app groups, BCVA in the amblyopic eye will be assessed as secondary outcome 1 and stereopsis in the amblyopic eye as secondary outcome 2. To assess the reliability and validity of stereopsis measurement using the VR app, secondary outcome 3 will involve comparing stereopsis in the amblyopic eye measured using the VR app for pediatric amblyopia with that obtained during clinical visits using the Titmus stereo test. To evaluate treatment adherence in the control and VR app groups, secondary outcome 4 will involve assessing the ratio of the total wearing time to the target wearing time. To evaluate the safety of amblyopia treatment using the VR app, secondary outcome 5 will involve comparing the angle of strabismus in the amblyopic eye between the control and the VR app groups. To investigate how VR gameplay status correlates with efficacy and safety, secondary outcome 6 will involve examining the VR gameplay status in the VR app group (including game type, play date or time, play duration, and game settings) in relation to BCVA, stereopsis, treatment adherence, and ocular deviation. Secondary outcome 7 will evaluate malfunctions of the VR app for pediatric amblyopia, secondary outcome 8 will monitor adverse events, and secondary outcome 9 will assess usability in the VR app group. Adverse events will be monitored throughout the trial. Based on our safety evaluation of the VR app in adults, where no visually induced motion sickness (VIMS) or vision-related adverse events occurred [18], the intervention is not expected to pose major safety concerns. However, careful monitoring remains essential in pediatric populations. Possible adverse events include VIMS, asthenopia, diplopia, transient blurring, and headaches. All adverse events reported by participants or observed during clinic visits will be documented and evaluated for severity and relationship with the intervention. Among the secondary outcomes, those related to BCVA, stereopsis, ocular deviation, and treatment adherence (secondary outcomes 1‐4) have been defined as key secondary end points. Since this is an exploratory clinical study, all secondary outcomes other than the primary outcome will be analyzed and interpreted in an exploratory (hypothesis-generating) manner.

### Sample Size and Statistical Analyses

The primary outcome of this study will be to determine whether the improvement in BCVA from enrollment to 12 weeks in the VR app group is noninferior to the improvement in BCVA from enrollment to 12 weeks in the control group. Based on the assumption that the difference in mean BCVA at 12 weeks between the control and VR app groups will be 0.07 LogMAR [35], the common standard deviation will be 0.14 [36] LogMAR, the noninferiority margin will be 0.10 LogMAR [37], the significance level will be 2.5%, and statistical power will be 90%, a sample size of 24 participants (12 per group) is required [19]. Anticipating a dropout rate of approximately 20%, the target sample size has been set to 30 participants (15 per group).

To assess whether the VR-based program for amblyopia is noninferior to eye patch therapy, the 95% CI for the change in BCVA between 0 and 12 weeks will be calculated for both the control and VR app groups. If the lower bound of the 95% CI for the change in BCVA in the VR app group is greater than that in the control group, the VR-based program for amblyopia will be considered noninferior to eye patch therapy, and the primary outcome criterion will be met.

For between-group comparisons of BCVA, stereopsis, ocular deviation, and the ratio of total treatment time to the target treatment time, 2-tailed *t* tests or repeated-measures mixed-effects models will be used. Within-group comparisons will be conducted with 2-tailed paired *t* tests, repeated-measures analysis of variance, or repeated-measures mixed-effects models. Reliability and validity evaluations of stereopsis measurements using the VR app compared with the Titmus stereo test administered at clinical visits will be performed using 2-tailed paired *t* tests, Cronbach α coefficients, or intraclass correlation coefficients. The associations between VR gameplay status, ophthalmological examination results, and treatment adherence will be examined using multiple regression analyses or mixed-effects models adjusted for confounding factors. The number of adverse events in the control and VR app groups will be compared using the chi-square test. If continuous variables deviate substantially from a normal distribution, more appropriate methods such as the Mann-Whitney *U* test will be applied. A 2-sided significance level of 5% (*P*<.05) and 95% CI will be used for all analyses. We will not apply multiplicity adjustments across secondary outcomes that represent different endpoint domains. However, when repeated measurements of the same outcome are compared at multiple time points (eg, longitudinal analyses of BCVA), appropriate multiple-comparison adjustments (eg, Bonferroni correction or equivalent family-wise error control) will be applied within that end point family. Primary and secondary analyses will follow intention-to-treat, including all data collected before withdrawal. A per-protocol analysis will be performed as supplementary to assess the robustness of the findings.

### Data Management

Data management will be performed by an independent external organization (Micron, Inc.) in accordance with a predetermined data management plan. Data will be collected and linked at multiple time points for each participant. Study investigators, including sponsor-affiliated authors, will not access study data before database lock. After lock, the final dataset will be transferred to an independent statistician within the external organization who has no financial or organizational relationship with the developer and will conduct all analyses. Because the external organization lacks medical expertise in pediatric amblyopia, investigators will help interpret the independently analyzed results and prepare the manuscript. The investigator group will make decisions about reporting and publishing study findings. However, once locked, the dataset cannot be modified; sponsor-affiliated authors cannot alter study data or analysis outputs. The final study report, including results and interpretation, will undergo review by the institutional ethics committee, and the manuscript will undergo independent peer review. These external oversight layers enhance transparency and safeguard research objectivity.

## Results

This study will begin on January 1, 2026, at 3 medical institutions in Japan. Patient enrollment will run from January 1, 2026, to September 30, 2026. The data analysis will begin on October 1, 2026, and the results will be reported by March 31, 2027.

## Discussion

Pediatric amblyopia can cause an irreversible reduction in visual acuity and has a profound impact on the quality of life. The conventional therapeutic approach, comprising full refractive correction and occlusion of the healthy eye, has established efficacy. However, the substantial psychological and physical burden imposed on affected children predisposes them to poor adherence and compromises treatment effectiveness, thereby undermining the overall outcomes of amblyopia therapy. Therefore, therapeutic modalities that minimize the burden on pediatric patients and concurrently enhance patient adherence and therapeutic efficacy in the treatment of amblyopia are warranted. This protocol paper outlines a multicenter clinical trial designed to evaluate the therapeutic efficacy of a VR app for pediatric amblyopia. The implementation of VR app–based amblyopia training has the potential to improve treatment outcomes by enhancing adherence through gamification and augmenting therapeutic efficacy via hand-eye coordination.

 VR-based digital therapeutics are increasingly recognized as promising therapeutic modalities for the management of pediatric amblyopia. In contrast to traditional treatment modalities, VR systems can independently modulate the visual input to each eye, thereby facilitating binocular training, which is regarded as critical in amblyopia therapy [38]. Furthermore, game-based amblyopia training that leverages gamification strategies to engage children’s interests is expected to enhance treatment adherence [39]. The objective of this study is to evaluate the therapeutic efficacy of a VR app for pediatric patients with amblyopia, utilizing the distinctive features of VR, in comparison with conventional occlusion therapy using an eye patch on the healthy eye. The VR app delivers game-based tasks that incorporate active bodily movements via hand-tracking controllers. The execution of these tasks necessitates hand-eye coordination. The combination of hand-eye coordination exercises with occlusion therapy in the healthy eye has the potential to augment visual recovery in the amblyopic eye through both direct visual stimulation and facilitation of neuroplasticity [40]. Unlike eye patch occlusion, which entirely blocks visual input to a healthy eye, this method permits simultaneous binocular training, offering the potential for greater visual improvement than conventional amblyopia treatments [41]. The demonstration of the efficacy of VR-based amblyopia training with a VR app in pediatric patients with amblyopia could introduce a novel therapeutic option that integrates the established benefits of occlusion therapy with improved adherence through gamification, promotion of neuroplasticity and visual recovery via hand-eye coordination, and additional visual gains through binocular training. Collectively, these features may drive a paradigm shift in amblyopia treatment.

Several studies have reported the efficacy of VR-based amblyopia training. One study incorporating the VR-based therapeutic device Luminopia One (Luminopia, Inc.) used a method wherein the contrast of streaming content presented to the healthy eye was reduced to encourage viewing with the amblyopic eye. Over a 12-week treatment period, significant improvements in visual acuity and stereopsis were observed, accompanied by high treatment adherence [19,42,43]. As an early example of a VR system, the Interactive Binocular Treatment system, a VR gaming platform employing a stereoscopic display and personal computer, was reported as early as 2006. Using this system, patients underwent treatment once or twice a week for approximately 20 min per session. In a cohort with a mean total treatment duration of 4.4 hours, an average improvement of 10 letters in logMAR visual acuity was achieved [44]. Additional VR-based approaches have been described, such as a photo-hunt game that requires the detection of inconsistencies between images presented to the 2 eyes [45] and NEIVATECH, a serious game–based system intended for binocular training in amblyopia that employs dichoptic presentation of images with differential contrast in a VR environment to implement perceptual learning tasks [41]. Each of these gaming systems seeks to achieve amblyopia training by projecting distinct visual stimuli onto the amblyopic and healthy eyes, thereby enhancing adherence through gamification. Contrastingly, the VR app for pediatric patients with amblyopia used in this study is intended to deliver, in addition to visual stimulation–based amblyopia training and binocular training akin to prior VR-based interventions, the promotion of neuroplasticity via tasks requiring hand-eye coordination. Accordingly, the VR-based SaMD for pediatric patients with amblyopia used in this study may yield superior therapeutic outcomes compared with conventional VR-based amblyopia training systems.

This study has certain limitations. First, despite the explicit inclusion criteria specifying participants with anisometropic, strabismic, or refractive amblyopia and a BCVA of ≤0.5 logMAR in the amblyopic eye, heterogeneity in disease severity, underlying etiology, and associated comorbidities may influence therapeutic outcomes. Second, interindividual differences in age, prior exposure to VR devices, and degree of familiarity may have affected both adherence and therapeutic response, especially during the initial phase of the study. To minimize the influence of such heterogeneity, participants allocated to the VR app group underwent an interim visit at week 2—prior to the first ophthalmologic assessment at week 4—during which VR app usage was reviewed to facilitate the proper continuation of treatment, even among participants with limited prior experience using VR systems. Third, the extended use of the VR app carries the potential risk of adverse effects, including VIMS and asthenopia. Our prior safety evaluation of the VR app in adults revealed no VIMS- or vision-related adverse events [18]. However, data on the long-term use of VR apps in pediatric populations remain limited, and the occurrence of adverse events could potentially affect treatment adherence and diminish the therapeutic efficacy of amblyopia management. Fourth, while amblyopia therapy in clinical settings is generally expected to extend beyond the 6-month duration of this study, this protocol does not permit the evaluation of long-term efficacy, posttreatment sustainability of therapeutic benefits, or the risk of amblyopia recurrence. Upon the completion of this study, an extension study is being conducted to allow interested participants to continue treatment with the VR app. The implementation of such a study with an extended follow-up would facilitate the assessment of long-term therapeutic efficacy and safety, persistence of treatment effects, and the likelihood of amblyopia recurrence. Fifth, the VR app–based stereopsis test used here was newly developed without formal validation. From a visual psychophysics perspective, this task uses a 4-alternative forced-choice design with size-constancy adjustments to reduce monocular cues. However, the extent of monocular cue elimination remains unverified. The floor and ceiling disparity levels of this stereotest are undetermined. Therefore, the stereopsis outcomes should be interpreted as preliminary. We included an in-headset stereopsis measure as it may provide a practical way to assess stereopsis under intervention conditions and enable monitoring between clinic visits, with potential utility for future clinical implementation and remote assessment in telemedicine.

In conclusion, a VR app may overcome the limitations of conventional amblyopia training by offering a highly effective therapeutic alternative, thereby facilitating a paradigm shift in amblyopia management. Future confirmatory clinical trials are required to delineate the efficacy and limitations of the VR app and to determine its potential integration into standard treatment strategies for pediatric amblyopia.

## Abbreviations

BCVA: best-corrected visual acuity
SaMD: software as a medical device
VIMS: visually induced motion sickness
VR: virtual reality
